# HIV-Associated TB Syndemic: A Growing Clinical Challenge Worldwide

**DOI:** 10.3389/fpubh.2015.00281

**Published:** 2015-12-23

**Authors:** Maria Theresa Montales, Arun Chaudhury, Alexandria Beebe, Sowmya Patil, Naveen Patil

**Affiliations:** ^1^University of Arkansas for Medical Sciences (UAMS), Little Rock, AR, USA; ^2^GIM Foundation, Little Rock, AR, USA; ^3^Arkansas Department of Health, Little Rock, AR, USA

**Keywords:** coinfections, extrapulmonary tuberculosis, HIV, TB, diagnosis, GeneXpert, AFB

## Abstract

The association of tuberculosis (TB) with human immunodeficiency virus (HIV) infection and acquired immune deficiency syndrome over the past several years has become an emerging syndemic. Approximately 10% of people living with HIV (PLHIV) with latent TB infection will develop active TB disease each year. In this review, we highlight that this phenomenon is not limited to high endemic regions, such as Afro-Asian nations, but globalization/migration is causing increased case detection even in developed nations, such as the United States. Active screening should be performed for TB in PLHIV. A high degree of clinical suspicion for TB is warranted in PLHIV presenting with fever, cough, and unintentional weight loss. HIV–*Mycobacterium tuberculosis* (MTB) coinfection is often paucibacillary, precluding diagnosis by conventional diagnostics and/or smear microscopy/culture. Improved detection of pulmonary and extrapulmonary TB is now possible by incorporation of the GeneXPERT MTB/RIF assay (Cepheid Inc., Sunnyvale, CA, USA). The World Health Organization recommends instituting immediate therapy for MTB, in conjunction with ongoing or newly introduced anti-retroviral therapy. Vigilance is required to detect drug-induced organ injuries, and early-treatment-induced immune reconstitution inflammatory syndrome. Collaborating MTB and HIV activities in concentrated HIV epidemic settings should become a high public health priority.

## Introduction

*Mycobacterium tuberculosis* (MTB) coinfection in patients pre-infected with human immunodeficiency virus (HIV) and/or with full-blown acquired immune deficiency syndrome (AIDS) is an emergent pandemic (1–3). Due to increased recognition of the morbidity and mortality associated with this coinfection, the World Health Organization (WHO) recommends aggressive approaches for MTB screening during primary visits related to HIV screening and treatment (2). While this state of coinfection is a major public health challenge in resource-constrained settings with a high burden of both diseases, such as those in African and Asian nations (1, 4, 5), it is being increasingly recognized in the settings of developed nations, including the United States (U.S.) (6–9). WHO estimated 1.5 million tuberculosis (TB) deaths in approximately 70% HIV-negative and 30% HIV-positive individuals, which makes MTB the second leading cause of death from an infectious disease and the leading cause of death in the setting of AIDS worldwide (10). This review presents the key clinical aspects of HIV–MTB coinfection, including the uncommon presentations, diagnostic issues, and management strategies, and emphasizes the continued need for increased vigilance for intensified case finding.

Per WHO guidelines, all clients attending HIV testing centers and people living with HIV (PLHIV) attending anti-retroviral therapy (ART) centers should be clinically screened for TB symptoms at every ambulatory encounter (2). Although the initiation rates of HIV-positive TB patients to ART are improving case fatality continues to be steep compared to HIV-negative TB patients (2). The chief rationale for this difference may include delays in bacteriological detection of HIV-associated TB, enrollment into ART care or immediacy of ART initiation (2).

Different populations pose group-specific challenges in response to detection and treatment. These populations include pediatric patients, antenatal HIV-infected patients, truckers, female sex workers, and men who have sex with men, refugees, and displaced populations (2). Other challenges exist in overcrowded settings such as mines, prisons, homeless shelters, and opioid substitution therapy centers (2). Social issues, including poverty, are warped into the fabric of clinical course of HIV–MTB coinfection (2). In this review, we emphasize early detection by effective implementation of provider-initiated HIV testing and counseling in TB patients as well as intensified TB case finding among PLHIV and initiation of prompt treatment to minimize morbidity and mortality (2).

## Demographic Factors Related to *Mycobacterium tuberculosis* Coinfection in HIV Patients

Gender, age, socioeconomic status, marital status, and educational level are factors that impact the likelihood of HIV–MTB coinfection. Male gender is reported to be positively correlated with MTB infection at a ratio of 2:1 (11). HIV–MTB coinfection is more common in adults with an average age of 33–45 years (2). Single status, low socioeconomic and income status, and lack of education are associated with disadvantaged living conditions. Compromised sanitation and poor access to healthcare negatively impact outcomes, which may explain the higher incidence and mortality in this group of patients (2).

It is important to outline some exceptions here. For example, young people may also be afflicted with the coinfection. Pediatric populations may particularly be at high risk, especially those who acquired infections by vertical transmission (12). Interestingly, certain geographic regions such as the Pakistan–Afghanistan border areas report more incidence in females than males (13). According to the WHO 2013 Global TB Report, Afghanistan is a high burden country for TB with a male to female ratio for TB of 0.5, so that in contrast to many other countries, Afghan women are more likely to be infected with TB than men. The estimated incidence of HIV–TB coinfections is relatively low (1 in 100,000) in this group and concentrated mostly among injection drug users (according to the World Bank). If there was a higher HIV–MTB coinfection incidence in Afghan women refugees, the observations would likely be due to the higher TB incidence in Afghan women or due to a small sample size of HIV–MTB coinfection in a refugee camp. Furthermore, immunocompromised states, such as those receiving anti-tumor necrosis factor treatment, corticosteroids, dialysis, organ, or hematologic transplantation or those with silicosis, may be more predisposed to HIV–MTB coinfection due to activation of latent TB infection (14).

## Pathogenesis of *Mycobacterium tuberculosis*–HIV Coinfection

The details of immune responses to TB, HIV, and coinfections have been described in recent reviews (1, 15). It is important to note that MTB occurs earlier in HIV patients than other opportunistic infections (OIs) due to increased susceptibility of MTB-specific CD4+ T-cells to HIV infection (16). We present the immune pathogenesis in relation to the intensity of the clinical presentation of TB with pre-existing HIV infection.

Tuberculosis infection is a result of the interplay between bacterial virulence and host resistance (17, 18). The infection begins through inhalation of air droplets containing approximately 1–200 bacilli from an individual with active MTB (pulmonary) disease (18). The bacilli are rapidly phagocytosed by resident macrophages in the alveoli. This triggers an inflammatory cascade, followed by development of granuloma. Furthermore, cell-mediated immunity through activation of CD4–T lymphocytes is important in the prevention of MTB disease’s acceleration and reactivation (1, 15, 17, 18).

On the other hand, HIV transmitted primarily through genital fluids, blood, and mucosa interacts with different cells in the body and tends to escape the host immune response against it, resulting in full-blown AIDS disease (19). Progression of HIV infection is a result of a combination of CD4+ T lymphocytes depletion and a chronic state of immune inactivation. The repression of CD4+ T cells and impairment of macrophages’ activity in HIV/AIDS results in down-regulation of the body’s immune response to infections, such as MTB. *Mycobacteria* are contained within granulomas; however, their disruption leads to MTB bacterial growth and systemic dissemination to multiple organs (1, 15). MTB has a negative impact on the immune response of the body to HIV by up-regulating the immune response of the host by activating T-cells. Studies have demonstrated that MTB enhances HIV viral replication by increasing the expression of receptors (e.g., CXCR4), which favors viral growth (15, 19). The immune response is responsible for the vigor of TB infection in a HIV-coinfected host and is responsible for miliary and extrapulmonary presentations and its associated diagnostic dilemma (1, 15, 20, 21).

Studies aiming to obtain direct evidence of disease progression have been limited due to economic reasons of HIV viral load estimation, especially in countries where the incidence/prevalence of HIV–MTB coinfection is high but has the shortcoming of resource constraints (22). As pointed out, the impact of MTB on HIV disease progression primarily involves upregulated HIV-1 viral load, including the development of new OIs (22–28). Interestingly, TB was found to exert significant effect on diminishing survival rates in subjects with more preserved immunological status (i.e., CD4 cell counts >200 cells/μL) (27, 28). Enhanced HIV-1 production has been demonstrated at local sites of MTB infection, for example, in bronchoalveolar lavage (BAL) fluid from TB involved, compared with uninvolved, lungs of patients with HIV-1/TB coinfection (29). A clinical presentation of TB that is particularly observed in HIV-1-infected patients is pleural TB, a common presentation in African coinfected patients (30, 31). These sites of active MTB infection act as foci of HIV replication and evolution of quasi-species, independent of systemic HIV-1 activity, and may be responsible for disseminated MTB infection in HIV-1 coinfected hosts.

*Mycobacterium tuberculosis* breaches the alveolar epithelium during the first phase of extrapulmonary dissemination. Molecular mechanisms for this cytolysis have been reported. For example, heparin-binding hemagglutinin adhesin (HBHA) facilitates MTB to bind to sulfated glycoconjugates on epithelial cells. Two gene products of the MTB RD1 gene, early secretory antigenic target 6 kDa (ESAT-6) and culture filtrate protein 10 kDa (CFP-10), have been causally linked to the cell lysis and extrapulmonary mycobacterial spread (32). The trafficking of mycobacteria to the regional lymph nodes, while essential for the development of a protective T-cell-mediated immune response, is the first extrapulmonary site of migration of MTB. Bacteria thereafter disseminated through the bloodstream and lymphatics lead to extrapulmonary tuberculosis (EPTB). Lung granulomas from MTB/HIV-1 coinfected patients release lower levels of *in situ* TNF production (33). Additionally, MTB–HIV-1 coinfected hosts have low circulating mannose-binding lectin levels (34). The complex interactions that take place between host T cells, Tregs, cytokine production, and overall impaired Th1 responses predispose to extrapulmonary infections in HIV-coinfected hosts (35, 36). Several primary immunodeficiencies, including Mendelian susceptibility to mycobacterial infections, enhance the overall risk of EPTB (37). These aspects merit detailed studies in the future.

## Screening for *Mycobacterium tuberculosis*–HIV Coinfection

World Health Organization formulated a policy on collaborative MTB/HIV activities with the chief aim to reduce the dual burden of MTB and HIV (2). WHO recommends MTB screening among HIV-positive patients at the time of diagnosis and before the initiation of ART. The screening for MTB should also be extended to the household contacts of HIV-positive patients (2). The screening tool utilizes a clinical symptom-based algorithm that consists of the absence or presence of current cough, fever, weight loss, or night sweats at the time of initial presentation and at every visit to health clinics. This tool can be used to identify patients who need further medical attention and those who need MTB chemoprophylaxis intended to prevent active TB disease (rather than prevention of TB infection *per se*).

## Diagnosis of *Mycobacterium tuberculosis* Infection in HIV-Coinfected Patients

A high degree of clinical suspicion and the need for obtaining a focused and detailed history merits emphasis. Numerous diseases may mimic presentations of TB, but TB should be high up on the differential for pneumonic presentations and HIV-associated thoracic diseases (7, 38). Initial screening with a tuberculin skin test (TST) or interferon-gamma release assays (IGRA) is recommended. TST, which can be affected by bacillus Calmette–Guérin (BCG) vaccination, and/or IGRA, which is unaffected by BCG vaccination, are routinely used screening methods for TB in asymptomatic, non-HIV-infected individuals in low TB prevalence nations. However, it may be noted that these tests have a “low specificity” (TST) or “have false positive and false negative results” (IGRA) and are of low diagnostic values in TB endemic areas, especially when there is coinfection with HIV. When there is a high degree of clinical suspicion or pneumatic presentations, the standard diagnosis of TB in symptomatic patients is typically not a TST or IGRA, but rather achieved with sputum smear microscopy. This is inexpensive, rapid, and easy to perform in a field setting but has a lower sensitivity in MTB–HIV coinfection. The sputum smear sample has the advantage of being used for culture of MTB and drug susceptibility testing. Because coinfection lowers microscopy sensitivity, several current diagnostic techniques are more sensitive than the conventional sputum smear microscopy in detecting MTB in HIV-positive individuals. A growth-based detection of MTB, in cultures and molecular techniques, such as nucleic acid amplification testing (NAAT), has been shown to be more sensitive and allows strain characterization and drug susceptibility tests (39, 40).

In 2013, the Food and Drug Administration (FDA) in the U.S. approved the GeneXpert MTB/RIF assay (Cepheid Inc., Sunnyvale, CA, USA), a NAAT-based diagnostic platform. WHO endorsed the use of this assay as the initial test for TB diagnosis in PLHIV or who are suspected of multidrug-resistant TB (MDR-TB) and has since been extensively used (41–44). The cost of the test, despite being subsidized, has been a barrier in many TB endemic countries to be used as a first line diagnostic tool.

**Figure 1:**
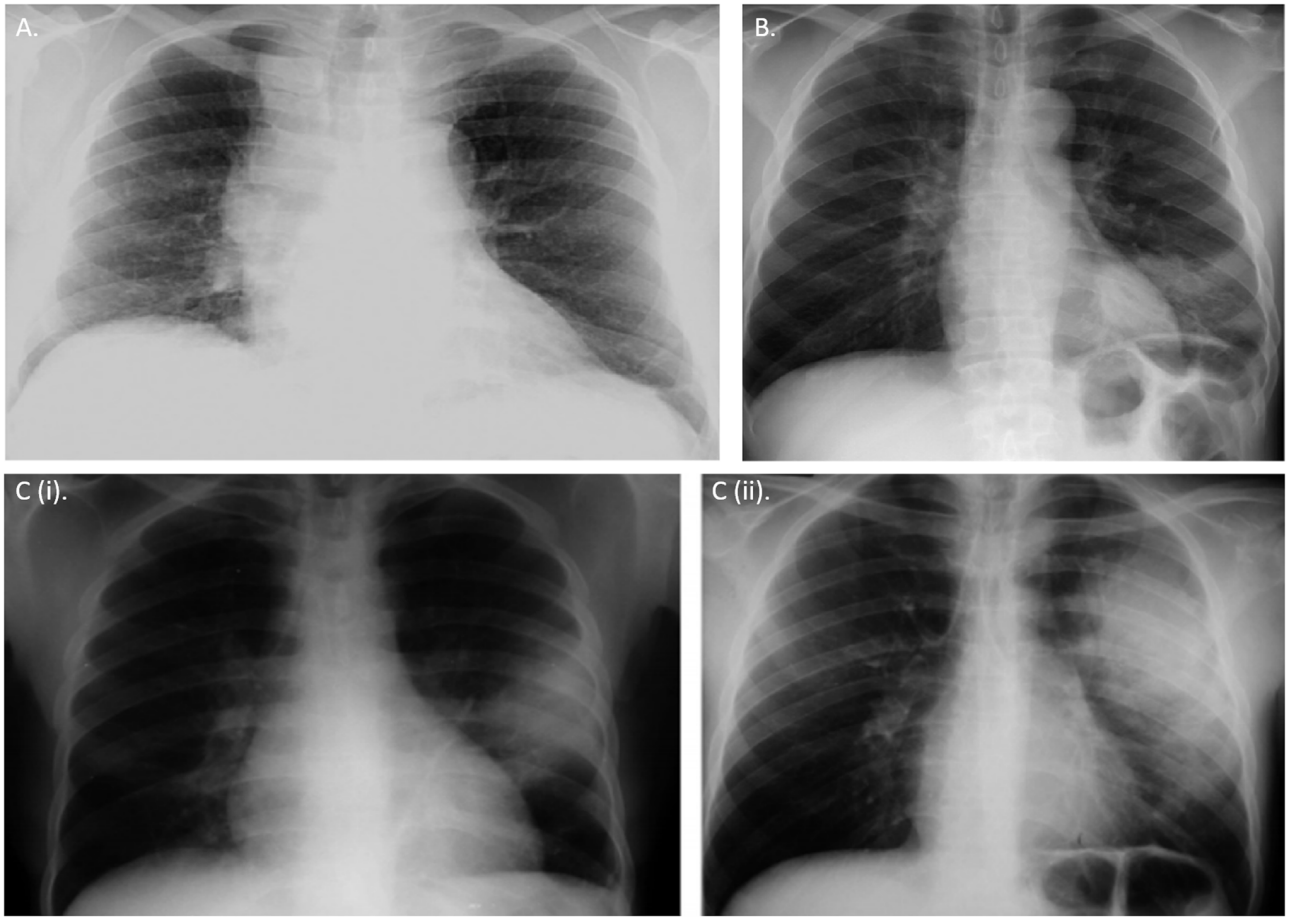
**Chest radiographic appearances of tuberculosis in HIV**. **(A)** Right hilar lymphadenopathy and bronchial markings in a primary pulmonary tuberculosis-like presentation in a HIV-infected young adult **(B)** Left lower lobe consolidation due to *Streptococcus pneumonia* infection in pre-existing tuberculosis and HIV coinfections **(Ci,ii)** *Radiologic appearance of IRIS*. Chest X-ray showing radiographic progression of a small left lobar lesion to widespread consolidation of a tuberculosis patient few weeks after initiating anti-retroviral therapy (ART). All CXRs obtained from permission with Chou et al. (38).

Additionally, low thresholds should be exercised when performing standard chest X rays and computerized tomography scans for diagnostic suspicion of miliary TB and detecting lesions during extrapulmonary manifestations (38). The pleomorphic manifestations of radiographic appearances in HIV–MTB coinfection is depicted in Figure 1.

A critical caveat in the diagnosis of MTB relates to the context of latent TB. The low specificity of TST and the issues of false positive and false negative detection with IGRA’s [QuantiIFERON^®^-TB Gold In-Tube (QFT-G-IT) assay (Cellestis Ltd., Carnegie, VIC, Australia) or T-SPOT^®^*TB* assay (Oxford Immunotec Inc., Marlborough, MA, USA)] pose significant challenges in HIV-coinfected patients. The critical aspects and unmet needs in diagnosing latent TB have been recently reviewed (39). Due to very high risk of activation of latent TB in HIV coinfection, these cohorts (immunocompromised subjects, immigrants from countries with high disease burden, homeless subjects, prisoners, illicit drug users, healthcare workers taking care of high risk group populations, and adult contacts of persons with TB) need careful identification and evaluation to be considered for follow-up, preventive therapy, and prompt institution of treatment upon detection of active lesions.

## Clinical Management of *Mycobacterium tuberculosis*–HIV Coinfection

Delay in the diagnosis of MTB in HIV-positive patients and inadequate initial treatment may lead to multidrug resistant TB (MDR-TB). MDR-TB is a growing concern not only because of its longer treatment duration but also because of its associated increased transmission risk among contacts and increased mortality rates in HIV-coinfected patients (45).

Considerations in the management of HIV–MTB coinfection include adjustment in the duration, dosage, and frequency of anti-TB drug administration as well as optimal timing of initiation of highly active anti-retroviral therapy (HAART) treatment. The standard therapeutic regimen for TB consists of isoniazid (INH), a rifamycin (rifampin-RIF or rifabutin-RFB), pyrazinamide (PZA), and ethambutol (EMB) given for 2 months, followed by INH plus rifamycin for 4–7 months (46). Treatment of active TB infection is the priority due to the risk of transmitting TB to other people. However, when CD4+ T cell count is extremely low (<50 cells/mm^3^), an appropriate treatment plan should be formulated wherein prompt initiation of HAART is necessary. Randomized controlled trials such as SAPiT (47), CAMELIA (48), and STRIDE (49) suggested and have provided evidence for immediate institution of ART within 2 weeks of starting anti-mycobacterial therapy.

There are several debates on the ideal timing of HAART initiation and concomitant administration with anti-TB medications. Advantages of early HAART administration include higher cure rates, reduced risk of relapse, reduced risk of infection with other HIV-associated OIs, and lower mortality rates. On the other hand, disadvantages include potential drug interactions of HAART with rifampicin, thus limiting co-administration of selected protease inhibitors (PIs), cumulative toxicity, therapeutic failure, and the risk of immune reconstitution inflammatory syndrome (IRIS), which affect the long-term adherence to HAART in MTB-infected patients (50). Furthermore, non-compliance due to pill burden, side effects of medications, accessibility of treatment centers for HIV and TB, fears of stigmatization, cost of healthcare, and lack of proper health education are also major problems that need to be addressed to successfully treat MTB patients (51). In general, the priority should be to provide directly observed therapy (DOT) for these patients to ensure compliance and treatment success (10). Two recently published trials have re-emphasized the importance of early initiation of ART. The TEMPRANO study from the Ivory Coast showed that early ART initiation in subjects with CD4+ cell count of ≥500 cells/mm^3^ was associated with a 44% lower risk of death or severe HIV-related illness than when ART was initiated according to prevailing WHO criteria (52). Furthermore, patients who received INH prophylaxis had a 35% lower risk of death or severe HIV-related illness than patients who did not receive it. In the START study involving 215 sites in 35 countries, the risk of death, a serious AIDS-related event, or a serious non-AIDS-related event was 57% less among the subjects treated early than among those treated when the CD4+ cell count decreased to 350 cells/mm^3^ (53). In both trials, a reduced rate of TB after early rather than deferred ART was the most significant contributor to the overall benefits. Early initiation of ART possesses the added benefits of reducing the risk of sexual transmission of HIV and HSV2 infections (54). However, the public health challenges concerning the cost and global health motivation for implementation of these recent evidence-based guidelines remains a major challenge that needs to be overcome (54).

In specific patients, such as HIV-infected pregnant women with active TB, the recommendation is to start on ART as early as feasible, both for maternal health and to prevent perinatal transmission of HIV (55). The choice of ART should be based on efficacy and safety in pregnancy and take into account potential drug interactions between anti-retrovirals and rifamycin (50). In HIV-infected patients with latent tuberculosis infection (LTBI), isoniazid preventive therapy (IPT) has been reported to reduce reactivation of latent TB infection in the context of both industrialized and developing countries (56).

## Complications Arising Out of Therapy for HIV–MTB Coinfection

### Immune Reconstitution Inflammatory Syndrome

Transient exacerbation of respiratory signs and symptoms despite reduction in viral load and/or radiological deterioration may develop in HIV–MTB coinfected patients who are treated with anti-TB medications concomitantly with HAART (19). IRIS has a dimorphic presentation: unmasking and paradoxical (57, 58). Restoration of immune competence by administration of ART results in hyperimmune host response to TB bacilli and/or antigens. Unmasking IRIS presents with active TB soon after ART is started. Paradoxical IRIS refers to the worsening of TB symptoms after ART is initiated in patients who are receiving TB treatment. Anti-inflammatory drugs and steroids are the mainstay therapy for IRIS. Discontinuation of HAART is not warranted in most cases. Delaying initiation of ART for 2–8 weeks may reduce the incidence and severity of IRIS. However, this possible advantage of delayed ART must be weighed against the potential benefit of earlier ART in improving immune function and preventing progression of HIV disease and mortality. Importantly, immune reconstitution with ART may result in unmasking LTBI (i.e., conversion of a previously negative TST to a positive TST or a positive IGRA for MTB-specific proteins) (44). A positive IGRA, similar to a positive TST, is indicative of LTBI in the absence of evidence of active TB disease. Because treatment for LTBI is indicated in the absence of evidence of active TB disease, this situation should be clinically recognized, especially in high risk groups.

### Anti-Tuberculosis and Anti-Retroviral Drug Interaction

Rifamycins are potent inducers of the hepatic cytochrome P (CYP) 450 enzyme. They are associated with significant interactions with all PIs, some non-nucleoside reverse transcriptase inhibitors (NNRTIs), maraviroc (MVC), and raltegravir (RAL) (50, 58–61), leading to enhanced drug clearance and significant lowering in circulating anti-retroviral drugs. However, good virological, immunological, and clinical outcomes may be achieved with standard doses of efavirenz (EFV) and nevirapine (NVP) when combined with rifampin (61). Suboptimal HIV suppression or suboptimal response to TB treatment should prompt immediate assessment of drug adherence, sub-therapeutic drug levels (with consideration for therapeutic drug monitoring), and acquired drug resistance.

### Other Known Side Effects

Hepatotoxicity potentially arises from co-administration of anti-retroviral and anti-mycobacterial agents; therefore, continuous monitoring of liver function should be exercised. Symptoms of abdominal pain, jaundice, loss of appetite, fever, and nausea merit urgent clinical attention (39). Additionally, many of these patients may need additional treatment, for example, for drug dependence or HCV coinfection, which presents additional risk for coexisting liver diseases (62). Peripheral neuropathy, on the other hand, can occur with administration of INH, didanosine (ddI), or stavudine (d4T) or may be a manifestation of the native HIV infection *per se*. All patients receiving INH should be administered supplemental pyridoxine to reduce peripheral neuropathy (61). Other coexisting medical and behavioral conditions, such as tobacco smoking, alcoholism, malnutrition, and diabetes mellitus, may significantly impact disease management and outcomes (63).

## Conclusion

Coinfection with the HIV is an important contributing factor to TB mortality not only in many countries, mainly those of sub-Saharan Africa, but also in developed countries such as the U.S. Today, caring for TB patients and controlling the spread of TB are complicated by the emergence of MDR-TB. Globalization and migration from endemic zones continues to be a major force in global spread of this coinfection. HIV-infected patients with active TB disease should receive treatment support, including adherence counseling and DOT, corresponding to their needs. In conclusion, MTB and HIV coinfection remains a diagnostic and therapeutic challenge worldwide.

## Conflict of Interest Statement

The authors declare that the research was conducted in the absence of any commercial or financial relationships that could be construed as a potential conflict of interest.
